# Viewpoints of community committee workers on public health emergency management in urban communities: a Q methodology study

**DOI:** 10.3389/fpubh.2026.1795922

**Published:** 2026-05-15

**Authors:** Hongling Shi, Wentao Li, Tong Yue, Xinxin Fan, Jiaxin Yu, Xue Wang, Libin An

**Affiliations:** 1 School of Nursing, Dalian University, Dalian, China; 2 Dalian Women and Children's Medical Center, Dalian, China

**Keywords:** community committee workers, public health emergency management, Q methodology, urban communities, viewpoints

## Abstract

**Objective:**

This study aimed to explore how community committee workers perceive public health emergency management capacity in urban communities and to identify structural weaknesses in current community-based emergency response systems.

**Methods:**

A Q methodology was employed to systematically examine community committee workers’ subjective viewpoints. A 35-statement Q set was developed based on semi-structured interviews and a literature review. Eighteen community committee workers were purposively recruited to rank these statements according to their level of agreement using a forced distribution ranging from −4 (strongly disagree) to +4 (strongly agree), followed by qualitative explanations of their rankings. Q-sort data were analyzed using Ken-Q Analysis to identify shared viewpoints.

**Results:**

Three distinct perspectives on community emergency management capacity were identified. Factor 1 emphasized resource support and coordinated execution, highlighting perceived constraints related to staffing, material support, and incentive mechanisms. Factor 2 focused on authority- and information-dependent response, reflecting participants’ concerns about fragmented information sharing and limited coordination across administrative levels. Factor 3 centered on risk communication and vulnerable populations, underscoring community committee workers perceived challenges in risk prevention for the older adults.

**Conclusion:**

Perceived limitations in community-level public health emergency management may be linked to structural misalignments within grassroots governance. These findings offer potential pathways for strengthening community emergency preparedness through resource support, coordinated information-sharing, and inclusive communication strategies.

## Introduction

In recent decades, the increasing frequency and complexity of public health emergencies have placed unprecedented strain on frontline public health systems, Urban communities, as the primary sites of population aggregation and the first points of policy implementation, play a decisive role in shaping the effectiveness of epidemic prevention and control efforts (1, 2). Their capacity to respond promptly and adaptively is therefore critical to the containment, mitigation, and recovery from public health crises.

Communities are entrusted with a wide range of responsibilities across the emergency management cycle, including prevention and emergency preparedness, monitoring and early warning, emergency management and rescue, and recovery and reconstruction (3). These responsibilities are examined through the perceptions of community committee workers, who interpret and enact them in practice. Acting as the closest interface between public health systems and residents, communities are often expected to function as the de facto “guardians” of population health. The effectiveness of these functions depends not only on formal institutional arrangements but also on how community-level actors understand, interpret, and carry out their operational responsibilities.

In this context, community resilience has emerged as a crucial mechanism for mitigating the long-term negative impacts of public health emergencies. While the academic definition of this concept encompasses multiple dimensions, a recent review by van Kessel et al. (4) indicates that community resilience fundamentally reflects a community’s ability to leverage its social, material, and economic resources to withstand and recover from health crises. As the “first line of defense” in public health emergency response, communities possess essential capabilities such as adaptation, self-organization, and transformation. Additionally, they harness unique local knowledge and social networks that effectively compensate for the shortcomings of top-down institutional responses. Integrating community resilience into health emergency management aligns with global strategies like the Sendai Framework for Disaster Risk Reduction (2015–2030) and the Health Emergency and Disaster Risk Management Framework (2019), marking a shift from reactive crisis response toward proactive preparedness and sustained recovery.

However, understanding community resilience requires moving beyond assessments of formal capacity and policy design. It also necessitates attention to how emergency management is perceived, interpreted, and enacted by frontline community committee workers in practice. Existing research on public health emergency management has generated substantial evidence. These literature has primarily focused on governance models and institutional innovation (5–7), the construction of evaluation indicators for emergency response capacity (3, 8), emergency preparedness and response capabilities (9), and the role of digitalization in enhancing grassroots governance (10). Notably, much of this literature adopts a macro-level, government-centered perspective (8, 11, 12).

With the advancement of community-level governance, scholars have increasingly acknowledged the importance of grassroots organizations in resource coordination, information dissemination, and the implementation of preventive measures (9, 12). However, less attention has been paid to how the capacities associated with community resilience, such as social capital, physical infrastructure, and health resources, are subjectively mobilized and operationalized in practice during public health emergencies (4). This limitation is particularly evident at the grassroots community level. Empirical research on public health emergency management remains limited, especially in capturing how community committee workers themselves perceive priorities, constraints, and responsibilities during emergency response processes.

This study adopts an inside-out perspective by centering the subjective viewpoints of frontline community committee workers. Their interpretations of policy directives, resource availability, and operational responsibilities directly influence how emergency measures are implemented on the ground (13), yet these perspectives remain insufficiently examined in the existing literature. Thus, the present study applies Q-methodology to explore the subjective viewpoints of frontline community committee workers regarding community emergency management capacity. Unlike conventional indicator-based assessments or variable-centered survey approaches, Q-methodology is specifically designed to identify shared patterns of subjectivity across individuals. This approach enables the systematic classification and comparison of distinct viewpoints, providing a methodological basis for understanding community resilience that complements existing macro-level governance analyses.

Dalian is a major coastal city in Northeast China with high population mobility, dense urban settlement, and extensive external economic exchange, creating a complex governance context for responding to public health emergencies. In addition, Dalian is China’s largest port cold-chain logistics base, which increases its exposure to infectious disease transmission risks, particularly through international trade and cold-chain pathways. In recent decades, Dalian has experienced multiple public health emergency events (14). Against this backdrop, this study took Dalian as an example to explore the structured subjective viewpoints of frontline community committee workers on community emergency management capacity. By capturing these latent viewpoints, the study aimed to provide empirical evidence that can inform differentiated community resilience-building strategies and complement existing macro-level governance frameworks.

## Methods

**Figure 1:**
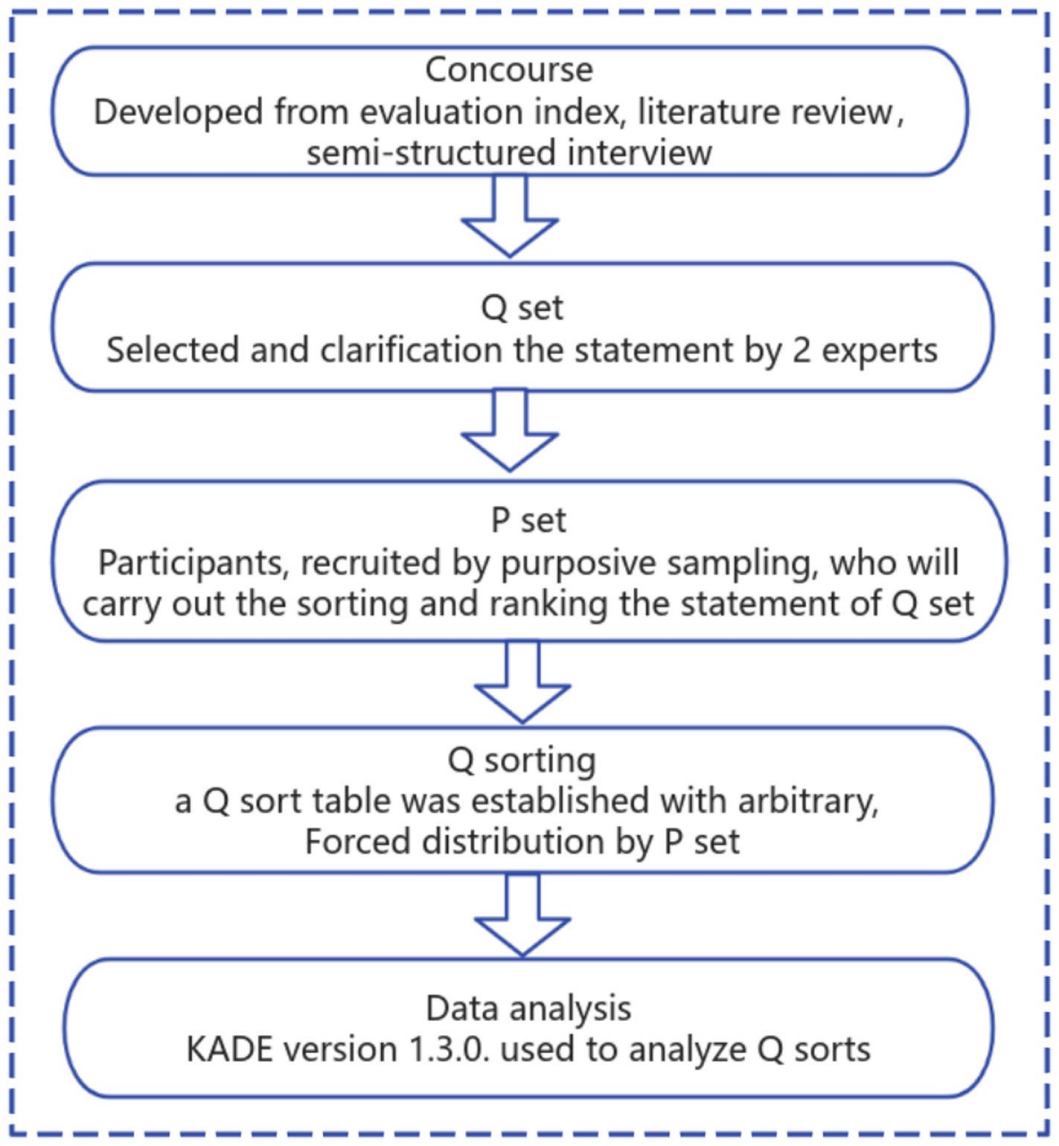
Study process.

Q-methodology, a form of by-person factor analysis proposed by British psychologist William Stephenson in 1935, is a scientific methodology used to study individual subjectivity and attitudes (15). This approach integrates quantitative and qualitative research methods, enabling the systematic transformation of subjective viewpoints into empirically interpretable outcomes (13). The methodology’s application has been described in nursing education and health research (16–18). Figure 1 illustrated the study process.

### Concourse and Q set

To build the concourse, data were collected using an evaluation indicator system for the emergency management capability of major infectious diseases (3), a literature review, semi-structured interviews.

The Q statement represents the main viewpoints related to the research topic. Twenty community committee workers from representative cities that had experienced infectious disease outbreaks were selected as interviewees: Dalian (*n* = 7), Wuhan (*n* = 7) and Guangzhou (*n* = 6). These cities constitute a group of frontline, high-density urban hubs in China that faced highly similar challenges during the pandemic, both in terms of public health pressures and the policy contexts they operated within. Inclusion criteria: (1) Having worked in the community for more than 5 years and being familiar with the community work; (2) voluntarily participating in this study. Each interview lasted no less than 30 min. During the interview, audio was recorded and non-verbal information was noted. All participants gave written informed consent before data collection. Subsequently, the recorded information was transcribed into text, and finally, a statement set relevant to the research purpose was determined. The interviews and transcriptions continued until information saturation was reached. This process initially generated 56 statements for the concourse. We confirmed data saturation through repeated responses across interviews. Two experts (professors of public health service management) were invited to examine and refine each statement in the Q statement to reduce ambiguity and eliminate redundancy. Finally, a set of 35 representative and important Q statements was generated. All interviews were conducted in neutral settings, maintaining privacy and ensuring confidentiality throughout the research process.

### P sample

The Q method aims to explore the participants’ interesting or important viewpoints on research topics. Unlike R-methodological surveys that require large representative samples for horizontal generalization, Q-methodology is characterized by the use of a small sample and focused on the diversity of representation rather than the sample of the population. Thus, participants in the P-set were recruited based on their relevance to the research topic. A P-set to Q-set ratio of approximately 2:1 is commonly recommended in Q methodology (19, 20). In this study, 18 community committee workers from Dalian, China were recruited as participants. They represented a range of community management experience, and all had direct involvement in frontline COVID-19 emergency response, thereby ensuring theoretical saturation and diverse representation in the data collected.

### Q sorting

**Figure 2:**
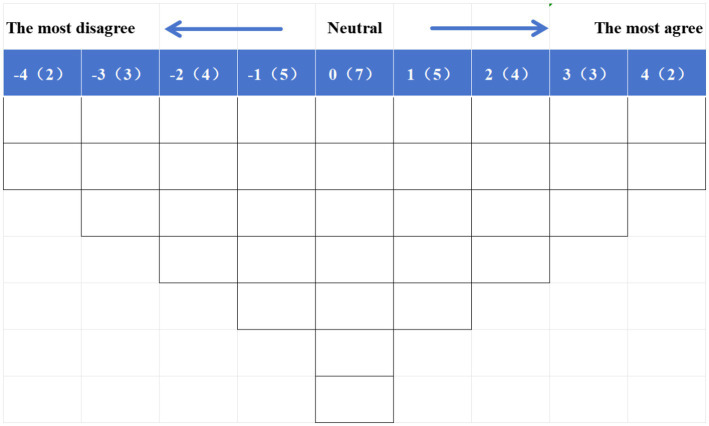
The Q sorting table with 35 grids for the current study.

A Q sorting table was developed with a nine-point scale ranging from “strongly disagree” (−4) through neutral (0) to “strongly agree” (+4). Participants in the P sample were asked to sort the Q set into disagreed, neutral, and agreed groups based on their subjective importance through a face to face, systematic forced distribution (Figure 2). Q-sort can be conducted using paper-based methods (21). After each Q-sort, participants were asked to provide explanations for the most agreed-upon and disagreed-upon statements. These explanations provided additional insight into participants’ viewpoints on the research topic.

### Data analysis

We analyzed the Q sort using the Ken-Q Analysis Desktop Edition (KADE 1.3.1). By-person factor analysis followed by varimax factor rotation was carried out for the data analysis. To extract and compare factors, standardized (Z) scores were computed to identify the representative characteristics of a factor from other factors, employing an eigenvalue of 1.0 or higher (22). Statements with a Z-score above +1.0 are considered positive views, while those below −1.0 are considered negative views (23). The percentage of variance explained by each factor, the cumulative variance explained by all factors, and the number of P sample in each factor were also computed. Factor interpretation was performed using factor arrays, which provide Z-scores for each statement. Both quantitative and qualitative data contributed to the depth and clarity of our interpretation.

## Results

### P sample characteristics

Eighteen participants of P sample with community committee workers completed the Q sorting process of 35 statements in the Q set. The characteristics of participant are presented in Table 1.

**Table 1:** Participant characteristics of P sample (*N* = 18).

Demographic		*n*	Percent (%)
Age (years)	33–39	7	38.9
	40–49	10	55.6
	50–59	1	5.6
Gender	Male	2	11.1
	Female	16	88.9
Education	Undergraduate	16	88.9
	Graduate	2	11.1
Years of work experience	1–5	3	16.7
	6–10	3	16.7
	11–15	9	50.0
	16–20	1	5.6
	>20	2	11.1

### Factor extraction and variance explained

The three factors explained 62% of the total variance, with Factor 1 accounting for 41%, Factor 2 for 14%, and Factor 3 for 7%. The eigenvalues for Factor 1, Factor 2, and Factor 3 were 7.4117, 2.5798, and 1.239, respectively. Thus, we computed the Factor 1, Factor 2, and Factor 3 into the varimax factor rotation with the total 62% of the variance. The correlation coefficients between factor 1 and factor 2, factor 1 and factor 3, and factor 2 and factor 3 were 0.1333, 0.5451, and 0.2024, respectively. The low correlations indicate that the three factors are relatively independent.

### Characteristics and labeling of each factor

Table 2 presents the factor scores alongside the corresponding Q-sort values for each factor. In this study, we named the viewpoints by Q-sort values and participant comments on the sorting process.

**Table 2:** Factor scores and Q-sort values of each factor.

No.	Q set	Factor 1		Factor 2		Factor 3	
		Z score	Q sort value	Z score	Q sort value	Z score	Q sort value
S1	Communities should pay attention to the information dynamics of infectious disease, and adjust the work focus timely.	−0.548	−1	1.308	3^*^	0.158	0
S2	Communities conduct emergency response based on the information of infectious diseases pushed by higher authority.	−1.022	−2^*^	1.712	4^*^	0.003	0^*^
S3	Communities conduct epidemiological surveys.	−0.335	−1	1.21	3^*^	−0.589	−1
S4	Communities should use big data to screen and capture infectious diseases information.	−1.732	−3	−1.887	−4^*^	−1.133	−2
S5	Communities use big data platforms to verify infectious disease information.	−1.421	−2	0.579	1	−1.133	−3
S6	Communities received infectious diseases information from higher-level authorities.	−0.635	−1	0.683	1	−0.137	0
S7	Communities uses a variety of ways to make residents pay attention to the infectious disease information.	0.798	1	−0.723	−2^*^	1.461	4^*^
S8	Communities enables the older adults to obtain timely information on the infectious diseases through various means.	0.197	0	−2.014	−4	1.431	3^*^
S9	Communities raises awareness among the older adults in a variety of ways to attach importance to infectious disease.	−0.203	0	−0.527	−1	1.385	3^*^
S10	Communities should concern the needs of the older adults.	0.448	0	−0.856	−2^*^	1.323	3^*^
S11	Communities should employ diverse approaches to ensure that the floating population acquire on infectious diseases information.	−0.285	−1	0.175	0	0.714	1^*^
S12	Communities should ensure an adequate supply and good performance of emergency equipment for infectious disease.	0.843	2	1.354	4	−0.298	−1^*^
S13	Communities regularly organizes training sessions on the utilization of emergency equipment for infectious disease.	0.398	0	0.728	1	0.272	1
S14	Communities has strengthened the routine management of supplies for infectious disease.	0.697	1	0.983	2	−0.893	−2
S15	Communities should be equipped with effective transportation vehicles (electric vehicles, bicycles, etc.) for infectious disease emergency management.	1.262	3	1.112	2	1.063	2
S16	Communities should be staffed with sufficient personnel specialized in the emergency management of infectious diseases.	1.388	4	−1.887	−4	0.744	1
S17	Communities should strengthen capacity to implement early warning and response measures.	0.952	2^*^	−1.053	−2	−0.460	−1
S18	Communities should be allocated special funds for emergency management from higher authority.	0.878	2	1.031	2	0.146	0
S19	Communities should update and revise the emergency plan to ensure its effective implementation.	0.658	1	−0.66	−1	0.861	2
S20	Communities should optimize the emergency management process for infectious diseases.	0.968	2	−0.301	0	0.475	1
S21	Communities should strengthen emergency drills for infectious disease.	0.701	1	−0.833	−2	1.610	4
S22	Communities should increase promotional, educational, and training activities regarding infectious diseases.	−0.047	0	−0.428	−1	−0.054	1
S23	Communities should publicize the risks of the occurrence and spread of infectious diseases.	−1.639	−3^*^	−0.052	0	−1.631	−3
S24	Communities is capable of assessing the risk of occurrence and spread of infectious diseases.	−1.765	−4^*^	−0.151	0	−0.430	−1
S25	Communities should pay attention to environmental sanitation.	−0.230	−1	1.007	2	0.281	1
S26	Communities focus on the key population at risk for infectious disease.	1.086	3	1.308	3^*^	−1.470	−3
S27	Communities should effectively share the information among various departments.	1.102	3^*^	−1.308	−3^*^	0.065	0
S28	Communities can effectively control the source of infectious diseases.	−0.650	−2^*^	0.271	0	−1.861	−3^*^
S29	Communities should establish rapid assessment process for infectious disease.	−0.902	−2	−0.655	−1	−1.903	−4^*^
S30	Communities possess the capability for rapid assessment of infectious disease.	−1.604	−3	0.225	0^*^	−1.903	−4
S31	Communities can identify and monitor high-risk groups of infectious disease.	−0.639	−2	0.504	1	−0.861	−2
S32	Communities should enhance collaborative efforts in the prevention and control of infectious disease.	0.616	0^*^	−0.602	−1	−0.033	0
S33	Communities should promote active participation from social organizations and residents in the prevention and control of infectious diseases through various forms.	1.090	3^*^	0.278	0	0.914	2
S34	Communities can effectively requisition infectious disease materials and provide certain compensation.	1.760	4	−1.059	−3	−0.633	−2
S35	Communities should provide a certain amount of funding subsidies for emergency management of infectious disease to personnel and volunteers.	1.100	3^*^	0.324	1	−0.589	−1

#### Factor 1: resource and coordination oriented viewpoint

Participants loading significantly on Factor 1 emphasized the importance of tangible resources, organizational capacity, and intersectoral coordination in community emergency management. This viewpoint strongly prioritized sufficient staffing specialized in infectious disease emergency management (S16, +4), the availability of effective transportation vehicles (S15, +3), and the capacity to requisition emergency materials with appropriate compensation mechanisms (S34, +4). Respondents associated with this factor also placed high value on strengthening interdepartmental information sharing (S27, +3, *p* < 0.05). In addition, they emphasized the participation of social organizations and residents (S33, +3, *p* < 0.05), as well as financial subsidies for personnel and volunteers (S35, +3, *p* < 0.05).

The most influential P sample (P7) in Factor 1 was a 44-year-old female who had an undergraduate education and over 20 years of work experience. She stated as follows: “Community committee workers undertake front-line prevention and control tasks, and appropriate subsidies can help improve their motivation.” Similarly, P9 was a 38-year-old female who had undergraduate education and 6–10 years of work experience. She added that “the community had 9 staff members serving over 20,000 residents, often working around the clock, and enduring a heavy workload*”*. P13 (40-year-old, female, 11–15 years of work experience) emphasized: “There should be incentive mechanisms for social organizations and volunteers, as otherwise it is difficult to sustain their engagement in the long term”.

In contrast, participants in this group expressed limited confidence in communities’ abilities related to risk assessment and communication. Statements regarding the community’s capacity to assess the risk of infectious disease occurrence and spread (S24, −4, *p* < 0.05) and to publicize transmission risks (S23, −3, *p* < 0.05) were ranked at the lowest positions. P11 explained that “The risk assessment of infectious disease involves various aspects, and they do not have the professional knowledge required to carry it out”.

Overall, Factor 1 reflects a viewpoint of community emergency management that prioritizes institutional support and coordinated execution.

#### Factor 2: authority and information dependent viewpoint

Factor 2 represents a viewpoint characterized by a strong reliance on information and directives from higher-level authorities. Participants associated with this factor agreed that communities should conduct emergency responses based on infectious disease information pushed by higher-level authorities (S2, +4, *p* < 0.05) and closely monitor information dynamics to adjust work priorities in a timely manner (S1, +3, *p* < 0.05). High rankings were also observed for conducting epidemiological investigations (S3, +3, *p* < 0.05), ensuring adequate supply and performance of emergency equipment (S12, +4), and focusing on key populations at risk (S26, +3, *p* < 0.05). P17 in Factor 2 was a 48-year-old female who has undergraduate education and 11–15 years of work experience. She explained: “We adjusted work priorities upon receiving the information regarding infectious disease from higher-level authorities”.

Conversely, this factor showed strong disagreement with statements related to community autonomy and technical capacity. The use of big data to screen infectious disease information was ranked lowest (S4, −4, *p* < 0.05), as was the need for sufficient specialized emergency personnel at the community level (S16, −4). Participants also assigned low importance to interdepartmental information sharing (S27, −3, *p* < 0.05). Participants believed that infectious disease information management within the community was fragmented and decentralized, which hinders comprehensive data sharing and efficient resource integration during the initial response. They expressed a concern that this fragmentation could delay the dissemination and timely availability of critical information. P10 stated as follows: “The technical capacity of grassroots communities is often limited, which hinders their ability to effectively screen and capture critical information.” P8 added: “The community communicates information through work groups or resident groups on WeChat. Since we cannot fully control all information, it would be preferable to have information shared across multiple departments.” Similarly, P12 said: “I have to deal with various forms required by different departments, which significantly affects my work efficiency.” Collectively, Factor 2 portrays community emergency management as a process primarily guided by top-down information flows and external authority rather than local capacity expansion.

#### Factor 3: community engagement and vulnerable population oriented viewpoint

Participants loading on Factor 3 emphasized community communication, public engagement, and support for vulnerable populations as central components of emergency management. This viewpoint assigned the highest priority to using multiple channels to disseminate infectious disease information to residents (S7, +4, *p* < 0.05), strengthening emergency drills (S21, +4), and improving access to timely information for older adults (S8, +3, *p* < 0.05). High rankings were also given to raising awareness among older adults (S9, +3, *p* < 0.05) and addressing their specific needs during public health emergencies (S10, +3, *p* < 0.05). P3 in Factor 3 was a 47-year-old female with undergraduate education and 11–15 years of work experience. She explained: “The older adults who are unable to use smartphones may not receive timely information on the prevention and control of public health emergencies.” P6 added: “As a vulnerable group, the older adults have weaker immune systems and a limited ability and low psychological resilience to cope with emergencies, which can easily worsen their condition and prolong recovery, so special attention should be given to the older adults” P15 said, “The floating population is unable to obtain information on public health emergencies, which increases the difficulty of prevention.”

In contrast, this factor expressed strong skepticism regarding communities’ technical and analytical capabilities. Statements related to establishing rapid assessment processes (S29, −4, *p* < 0.05), possessing rapid assessment capacity (S30, −4), and effectively controlling sources of infectious diseases (S28, −3, *p* < 0.05) were ranked at the lowest levels. Participants in Factor 3 reported that it was difficult for them to conduct rapid assessments, issue emergency warnings, and provide early intervention for high-risk populations because of limited professional knowledge and skills. P18 explained that “The community merely executes tasks related to the prevention of public health emergencies as directed by higher authorities, and lacks the capability to assess risk and control the source of infection.” This viewpoint thus frames the community primarily as a hub for risk communication and social support rather than as a locus of technical assessment or disease control.

## Discussion

This study identified three distinct viewpoints among frontline community committee workers regarding community emergency management capacity, reflecting heterogeneous understandings of how communities contribute to public health emergency response. Rather than representing different levels of capacity, these viewpoints reveal divergent conceptualizations of community roles, priorities, and functional boundaries within the emergency management system. By foregrounding frontline subjectivity, these findings provide an inside-out explanation for why community committee workers perceive that community-based emergency responses may remain constrained despite substantial institutional attention and policy investment. Moreover, they advance the concept of community resilience by demonstrating that resilience depends not only on the provision of institutional, material, and informational resources, but also on the ways in which community committee workers perceive, interpret, and enact their roles in practice.

While communities are expected to function as frontline responders, coordinators, and “sentinels,” participants highlighted a perceived lack of decision-making power and professional autonomy. Adequate resource availability is widely recognized as a foundational prerequisite for effective community-level emergency response in public health crises. Previous studies have consistently shown that shortages in human resources, materials, and logistical support constrain frontline response capacity and undermine the sustainability of emergency operations, particularly in resource-limited settings (24–26). Factor 1 in our study emphasized the importance of staffing adequacy, material reserves, transportation capacity, financial incentives, and interdepartmental information sharing. Together, these elements appear to reflect how participants perceived their ability to mobilize tangible resources in a timely and coordinated manner. Importantly, our findings suggest the presence of what participants described as a persistent structural dilemma of “heavy responsibilities with limited authority” in community emergency management. Participants in this study observed that, in practice, existing mobilization models tend to rely on administrative mandates or moral appeals, whereas sustainable compensation and incentive mechanisms are perceived as insufficient. This finding is aligning findings from other low- and middle-income countries (27–29). These viewpoints suggest that frontline workers view communities as operational hubs whose effectiveness is rigidly constrained by structural inputs. Within the framework of community resilience, this finding aligns with the institutional and infrastructural dimensions, highlighting the importance of sustained resource investment and coordination mechanisms for maintaining operational continuity during public health emergencies.

Effective public health emergency response depends not only on the availability of resources but also on timely, accurate, and bidirectional information flows. Prior research has shown that fragmented information systems and weak coordination across administrative levels undermine situational awareness and delay frontline responses, particularly in resource-constrained contexts (30). Participants in this study perceived that community-level emergency actions were largely triggered by information pushed from higher-level departments. This pattern appears to reflect a government-dominated model of emergency governance, while simultaneously revealing the passive role of grassroots communities in information acquisition and interpretation. The resulting delays and fragmentation in information dissemination are not solely attributable to technical limitations. More fundamentally, they reflect the absence of stable collaborative mechanisms that support horizontal information exchange and local-level synthesis. Similar challenges in health information sharing have been documented in previous studies, especially where interdepartmental coordination remains weak (30). Limited information exchange further constrains communities’ capacity to support timely decision-making, effective resource allocation, and continuous outbreak monitoring (31). These findings suggest that strengthening community-level emergency governance requires more than improving information delivery. Integrated information systems must be accompanied by enhanced local analytical capacity and collaborative platforms, enabling communities to complement top-down directives with context-specific assessment and response (12).

Technology-enabled governance has become a central strategy in public health emergency management, yet evidence suggests that such approaches may inadvertently exacerbate existing social inequities. Prior studies have shown that unequal access to digital tools and information can increase exposure risks among vulnerable populations, particularly older adults and migrant workers (32, 33). Consistent with this concern, Factor 3 in our study highlights the digital divide and social inequities emerging under technology-driven emergency governance. Participants emphasized that high-risk populations, especially older adults, faced disproportionate challenges in risk prevention due to limited digital literacy, restricted access to digital platforms, and age-related physiological vulnerability. Barriers to accessing and interpreting risk information further undermine communities’ ability to identify and prioritize the needs of these populations. When risk communication relies heavily on digital channels, community committee workers encounter difficulties in accurately assessing exposure risks and tailoring timely interventions for older adults, thereby reducing the relevance and effectiveness of preventive services (34). These findings indicate that technology-centered emergency management, if not accompanied by inclusive design and alternative communication pathways, may lead to risk prevention failures among socially vulnerable groups. Addressing this challenge requires integrating equity considerations into digital governance frameworks to ensure that technological efficiency does not come at the expense of population-level inclusiveness.

Across all three factors, a notable and consistent pattern emerged: a pronounced lack of confidence among community committee workers in their own professional and technical capacities. Participants reported that risk assessment and control of infection source were repeatedly identified as the least developed competencies. This shared perception reflects a broader recognition that communities lack the specialized expertise needed to conduct risk assessments and implement preventative or mitigative measures to avoid risks both before and during a health emergency event. Community committee workers largely viewed themselves as implementers of directives issued by higher-level authorities, rather than as autonomous actors in public health decision-making. While the previous evaluation index and community resilience theory regard communities as frontline “sentinels” for early detection and risk monitoring (3, 4), participants felt that the professional capacity necessary to fulfill this role has not been correspondingly established at the grassroots community. They emphasized that the absence of personnel with formal public health training further constrains their ability to assume evaluative and analytical functions. These findings suggest that future capacity-building efforts should move beyond simply expanding community responsibilities. Clear delineation of functional boundaries may therefore help communities focus on screening, reporting, and service delivery, while professional risk assessment functions are either centralized at higher levels or supported through the deployment of trained public health professionals to the community level.

### Limitations

Several limitations should be considered when interpreting the findings of this study. First, Q-methodology is intended to identify shared patterns of subjectivity rather than to estimate their prevalence in a wider population. The identified factors should not be interpreted as representative distributions among all community committee workers, but rather understood as distinct patterns of shared perception within the sampled group. Second, while the sample size conforms to methodological standards for Q-methodology, it remains relatively small and geographically confined. Participants were recruited from urban community committee workers in Dalian, an urban setting with its own demographic profile, and public health emergency management experience. These features give the findings analytical relevance for other cities with similar institutional and social conditions; however, they may not be directly transferable to rural settings or regions with significantly different contexts. Third, the findings only reflect the viewpoints of community committee workers. Public emergency health response also requires collaboration among multi-stakeholders. Future research should examine urban samples across diverse socioeconomic contexts and incorporate the perspectives of health administrators, public health experts, and community residents, especially vulnerable groups, to evaluate the stability and practical relevance of these viewpoints.

### Implications

The findings of this study suggest that improving community responses to public health emergencies may benefit from considering structural misalignments within grassroots governance. First, strengthening resource and incentive arrangements offers a potential pathway to enhance the sustainability of emergency response. In particular, more stable staffing, material support, transportation resources, and appropriately designed incentive mechanisms are important for community committee workers’ response to sudden public health crises. Second, improving community-level response may depend not only on faster information delivery, but also on whether community committee workers are able to interpret, share, and act on that information in a coordinated manner. In such settings, integrated data-sharing systems and clearer interdepartmental coordination mechanisms could mitigate information fragmentation. Third, leveraging digital technology alongside offline communication facilitates the creation of smoother channels through which community committee workers can respond to residents’ needs, concerns, and service demands.

## Conclusion

This study provides an in-depth examination of how frontline community committee workers perceive community capacity in responding to public health emergencies, revealing structured and differentiated viewpoints. More importantly, the findings suggest the presence of perceived structural misalignments in grassroots communities’ responses to public health emergencies: expanded responsibilities clashing with limited authority, insufficiently integrated information systems, and challenges in ensuring equitable support for vulnerable populations. From a community resilience perspective, improving community emergency response may involve stable resource and incentive arrangements, better integrated data-sharing and interdepartmental coordination mechanisms, and inclusive communication strategies that combine digital technology with offline engagement. Future research should extend this line of inquiry by incorporating multi-stakeholders perspectives and longitudinal designs to examine how community roles, capacities, and governance relationships evolve across different phases of public health emergencies. Such efforts would further contribute to the development of resilient, equitable, and sustainable community-based emergency management systems.

## Data Availability

The original contributions presented in the study are included in the article/Supplementary material, further inquiries can be directed to the corresponding author.
