# Supplementary material for: Viewpoints of community committee workers on public health emergency management in urban communities: a Q methodology study
Source: Front Public Health. 2026 May 15;14:1795922. doi: 10.3389/fpubh.2026.1795922 (PMC13219005; doi:10.3389/fpubh.2026.1795922)
Supplement: Supplementary file 1 [file Table_1.DOCX]

Suplementary Material 1: Interview Outline

(1) What is your evaluation of your community's ability to process information related to infectious disease prevention and control?

(2) What problems come up when you try to send warnings and information regarding infectious diseases to individuals in your community?

(3) What enhancements are necessary concerning the quantity and types of equipment for the prevention and control of infectious diseases, as well as the mechanisms for emergency response?

(4) What is your evaluation of the community's command and coordination capabilities in the prevention and control of infectious diseases?

(5) What types of recompense has the community provided to residents for materials and labor that were removed following an infectious disease outbreak?

(6) Is the community capable of performing effective risk assessments for infectious diseases?

(7) Do you know how to deal with infectious disease emergencies? What would you do first?

(8) How would you evaluate the practicality of the infectious disease emergency plan in the community?

(9) What emergency drills has the community planned in preparation for a sudden outbreak of infectious diseases?

(10) Has your community implemented a rapid assessment workflow for the infectious diseases?

(11) What dilemmas and constraining factors currently exist in community emergency management regarding major infectious diseases?
